# Blessings and Curses: Exploring the Experiences of New Mothers during the COVID-19 Pandemic

**DOI:** 10.3390/nursrep10020023

**Published:** 2020-12-21

**Authors:** Phillip Joy, Megan Aston, Sheri Price, Meaghan Sim, Rachel Ollivier, Britney Benoit, Neda Akbari-Nassaji, Damilola Iduye

**Affiliations:** 1 Department of Applied Human Nutrition, Mount Saint Vincent University, Halifax, NS B3M 2J6, Canada; 2 School of Nursing, Dalhousie University, Halifax, NS B3H 4R2, Canada; megan.aston@dal.ca (M.A.); sheri.price@iwk.nshealth.ca (S.P.); meaghan.sim@dal.ca (M.S.); rachel.ollivier@dal.ca (R.O.); damilola.iduye@dal.ca (D.I.); 3 Rankin School of Nursing, St. Francis Xavier University, Antigonish, NS B2G 2W5, Canada; bbenoit@stfx.ca; 4 School of Nursing, Abadan Faculty of Medical Sciences, Abadan, Iran; neda.akbari@dal.ca

**Keywords:** family, bonding, COVID-19, mother, post-partum

## Abstract

The aim of this study was to explore the postpartum experiences of new parents during the COVID-19 pandemic. The postpartum period can be a time of significant transition, both positive and negative, for parents as they navigate new relationships with their babies and shifts in family dynamics. Physical distancing requirements mandated by public health orders during the COVID-19 pandemic had the potential to create even more stress for parents with a newborn. Examining personal experiences would provide health care professionals with information to help guide support during significant isolation. Feminist poststructuralism guided the qualitative research process. Sixty-eight new mothers completed an open-ended on-line survey. Responses were analyzed using discourse analysis to examine the beliefs, values, and practices of the participants relating to their family experiences during the pandemic period. It was found that pandemic isolation was a time of complexity with both ‘blessings and curses’. Participants reported that it was a time for family bonding and enjoyment of being a new parent without the usual expectations. It was also a time of missed opportunities as they were not able to share milestones and memories with extended family. Caring for a newborn during the COVID-19 pandemic where complex contradictions were constructed by competing social discourses created difficult dichotomies for families. In acknowledging the complex experiences of mothers during COVID-19 isolation, nurses and midwives can come to understand and help new parents to focus on the blessings of this time while acknowledging the curses.

## 1. Introduction

The postpartum period is often a complex time for new parents. Social, cultural, and medical discourses have created norms and shaped the beliefs, values, and practices of new parents during this time. These discourses position the postpartum period as a time for new parents to bond and form connections not only with their baby but within their larger family. Postpartum is well acknowledged as an opportunity for family-forming [1]. Social, health, and nursing discourses of mother-child bonding are dominant during this period and positions such bonding as critical for the development of healthy relationships, positive mental health and confident parents [2,3].

Family health nursing is a specialty clinical practice that focuses on understanding how relations within the family impact their health [4,5]. Decades of research have demonstrated the need to understand the family as a unit where “…family has a significant impact on the health and well-being of individual members (p. 1)” [6]. However, the family does not operate in isolation and supportive relations with extended family, friends, health care professionals, and others are extremely important [6,7]. 

The postpartum period is also a time for much needed social support [2]. New parents often seek and need social support for a variety of reasons from extended family members, friends, other parents outside their immediate social networks, nurses, midwives, lactation consultants, and other health care professionals. It is a time when new parents learn about their babies and when new families adapt to their changing experiences and the expectations placed upon them. Social discourses about the role of mothers perpetuates knowledge about what it means to be a mother and (re)creates many expectations for them [8,9]. New parents are challenged to balance new parental roles, work, extended family relations, and a myriad of other responsibilities while also attempting to enjoy these early moments with their baby [10]. 

Postpartum is also a period of time in which new parents are tasked to ensure they start their babies off onto the “right” path to be successful, whether this involves creating loving moments of family bonding, socialization with their babies, or ensuring proper nutrition [9]. Emotional and social well-being of infants and children are significant parts of these expectations. In the field of psychology, research continues to examine the emotional and psychological development of children and correlations with socialization and behaviour. Brownell [11] states: “…socialization of prosocial behavior occurs continuously via social engagement beginning at birth. Because the infant participates actively and eagerly in social and emotional exchanges, socialization encompasses more than top-down teaching or shaping processes and selected social-learning processes such as imitation. Instead, socialization includes many bidirectional social processes, some of which are quite subtle (p. 223)”. 

Furthermore, social networking for new parents is one way to seek out relationships with other parents to share information, experience support and gain confidence in their parenting abilities with their newborns [12,13,14,15,16,17]. Social networking occurs both online and offline with the purpose of facilitating supportive meeting spaces for babies and parents. Research has identified that peer and social supports are essential in the postpartum period to improve outcomes such as breastfeeding or maternal mental health, including postpartum depression [18,19,20,21]. 

Isolation can put parents at risk for mental health issues; therefore, connections between people can help to alleviate some of the risks. Postpartum programs and services offered by health care professional and community groups all focus on supporting the physical, emotional, and social well-being of parents. Our research to date has demonstrated that social networking is an essential part of the postpartum period [22,23] and is important for both parents and babies to ensure healthy short term and long term mental and emotional health. The COVID-19 pandemic has significantly reduced opportunities for parents to gather or meet with family, friends, other parents and health care professionals [16,24,25,26]. Therefore, examining the postpartum experiences of parents during the COVID-19 pandemic and required self-isolation, enabled us to examine how parents in Nova Scotia coped with the social and relational aspects of postpartum.

The overarching aim of this study was to examine parents’ experiences of the postpartum period during the mandated health protection orders in response to the COVID-19 pandemic. The research also explored how various social and institutional discourses shaped their experiences. The research question was ‘How do parents experience the postpartum period during COVID-19′?

## 2. Experimental Section

### 2.1. Theoretical Framework

Feminist poststructuralism was used as the guiding methodology [27,28,29,30,31,32] as it provided a way to understand how experiences were personally, socially and institutionally constructed through different subject positions. A feminist poststructuralist methodology allowed us to look for moments of negotiation to understand how different beliefs, values, and practices were constructed through relations of power between people [27,28,29,30,31,32]. The concept of subjectivity enabled us to examine how participants felt in relation to others (health professionals, family or peers). The concept of agency guided our analysis to consider how all individuals have power and therefore the potential to control their lives and make change [27,28,29,30,31,32]. Feminist poststructuralist methodology is based on the belief that participants are the primary experts of their experiences, and therefore are credible sources of data who are self-reflexive, conscious of their own locations (social, historical, gendered, cultural, racial, sexual), able to question, challenge, and possibly change their own circumstances. They also have the potential to recognize the oppressive nature of social structures, stereotypes, and ideologies. The study employed the Standards for Reporting Qualitative Research (SRQR) [33]. 

### 2.2. The Researchers

The team consists of nursing and health experts in the area of maternal child and infant health, public health nursing, women’s, family, and community health. Members of the team use qualitative methodologies, in particular feminist poststructuralism to explore how the health practices of individuals and families in modern society are shaped by historical contexts. Several members are registered nurses within Canada. 

### 2.3. Context of the Study

The study took place in Nova Scotia, Canada. Approval for the study was received through the IWK Health Centre’s Research Ethics Board (#1025663). Data was collected from May to June 2020, which represented the emergence of the recovery period of the COVID-19 pandemic in Nova Scotia, Canada. The peak COVID-19 or first wave of the COV-19 pandemic period was March and April 2020. During this time, the provincial public health measures were implemented within the province. The public health order was for households to stay physically distanced from one another. This requirement was from March to May, at which point the bubble family was introduced (early May), followed by small groups of less than 10 outdoors. Self-isolation (or isolation/quarantine) was much more restrictive and for certain persons/families, such as those that travelled outside the province. Following the public health orders, travel was restricted, and many workplaces were either closed or employees were instructed to work from home, if possible. Many retail stores and resources for new mothers, such as daycares, family resource centres, and public libraries, were also closed, although grocery stores remained open. Hospital services were restricted, and many consultations were done virtually (via phone or through the use of other technologies) rather than in person [34]. All of these factors led to periods of isolation experienced by the parents/families in this study and the inability to access their usual support networks.

### 2.4. The Qualitative Survey and Participants

Parents (biological, adoptive, foster, kin) who self-identified as the primary caretakers of a newborn baby aged 0–12 months during the time of the COVID-19 pandemic (beginning March 2020) were recruited through social media recruitment to participate in an on-line open ended survey. Participants were encouraged to write as much as they wanted to describe their experiences within their families, as well as any supports they may have had during this period. Specifically, three opened ended questions were asked. These questions were (1) Tell us about your experience at home with your new baby and how the situation created by the COVID-19 pandemic affected you and your family; (2) Tell us about your experience of support (from friends, family, healthcare professionals) during the COVID-19 pandemic; and (3) Tell us about your experience of searching for and receiving information about caring for yourself, your baby, and your family during the COVID-19 pandemic. Responses varied in length. 

We recruited 68 participants to complete the qualitative survey. All participants lived in the province of Nova Scotia in Canada, with over two thirds of them living in cities or towns in the province. Although we purposefully put out a call to include a variety of parents/guardians, all participants self-identified as mothers who gave birth within the last year and predominantly identified as heterosexual, white women. Most participants were living with their partners, with only a few identifying as a single parent. Approximately half of the participants reported having other children in their care during this time.

### 2.5. Data Analysis

Discourse analysis was used to analyze the data. Consistent with the feminist poststructuralist approach of the research, the use of discourse analysis [29] enabled the meaning of personal experiences to be deconstructed as a means of exploring how they related to social and institutional beliefs, values and practices. Discourse analysis is a non-linear process that attempts to look beyond the surface meanings of text to situate them within historical, political, social, and cultural contexts [29]. For this process, we paid close attention to language, meaning and relationships between participants, others and the health care system. All researchers independently reviewed the participants’ responses and noted the beliefs, values, and practices of the participants relating to their family experiences during the pandemic period, the language of the participants, as well as any tensions expressed. The team met as a whole to discuss the independent analysis and came to a consensus on the final discursive considerations.

Several key characteristics, such as rigor, trustworthiness, and credibility were attained through various processes. For example, the maintenance of accurate documentation, an audit trail that included detailed notes on the way responses were analyzed by each team member, positionality and reflections of researchers, and notes that recorded the ongoing discussions between the team during the analysis ensured rigor, trustworthiness, and creditability [35]. 

## 3. Results

This research revealed that public health orders due to COVID-19 pandemic created situations where the majority of parents had to negotiate complex family relationships that were often expressed as a duality between positive (blessings) and negative (curses) experiences. The period of isolation during COVID-19 was seen as both emotionally stressful and emotionally rewarding for many of the participants. Most participants responded with personal examples of the complexity of experiences, their comments discussing what they enjoyed during the postpartum period, while also moving on to discuss the tensions and stresses of their experiences. 

The nature of qualitative analysis is often complex and not easily separated into distinct sections. As such, we present in this section not only the results but also the analysis of the associated social discourses. The first Section 3.1 examines the construction of positive experiences/ “blessings” through discourses and the second Section 3.2 examines the construction of challenges/ “curses” through discourses. Participants’ quotes are used throughout to represent the discursive considerations.

### 3.1. The Blessings

In this study, we define blessings as a beneficial thing for which one is grateful and something that brings well-being. The following section explores the various positive blessings that the participants perceived from their experiences during COVID-19. The described blessings that resulted from following public health orders were multi-faceted and took many forms in the data. 

### 3.2. The Blessings of Freedom

One such blessing was participants’ freedom to enjoy their babies. A common experience for many mothers was the feeling of freedom from social expectations, including fulfilling the dominant socially constructed roles and identities set before many new moms, as illustrated in the following quote.

COVID has been a blessing and a nightmare for our new family… [our baby] gets a lot more daddy time every day and I get help during the day if I need it… I now feel no pressure to be a “super mom”. I just focus on spending time with her [baby] and enjoying her baby days. I know if the pandemic hadn’t happened, I’d be out doing “mommy and me” classes, doing visits, and generally trying to be more productive.

The requirement for families to stay within their own household was seen as a blessing for many of the participants because it created a sense of freedom from the social expectations placed upon new mothers to engage in “productive”, externally-focused activities, like mommy classes, visits from family members and friends, and other activities. Instead of feeling obligated by societal pressures and a cultural discourse of productivity to participate in new mother activities and obligations, several participants were able to find a relief from these pressures. 

It is interesting to look at how the participant in the quote above constructed the word ‘productivity’ outside of the home. This dichotomy has been created through a cultural discourse of productivity that gives more respect to work outside the family or household unit [36]. Parenting has been socially constructed to be less visible and less respected compared to work outside the home, an issue that is highly influenced by the gendered norms within society that also work to devalue emotional labour [36]. This socially constructed meaning about caring for a new baby often leads to unrealistic expectations for mothers to ‘do it all’ while caring for their children [36]. Previous research has shown that pressures to do the right things, and to be a “super mommy” can add a lot of pressure and stress to the experiences of new motherhood [8,9]. Many of the participants in our study expressed similar experiences of feeling less pressure to have to do it all. One participant said, “I found it great to bond as a family” and another stated “spending a lot of time doing things together…relaxed and no pressure to go anywhere”.

### 3.3. The Blessings of Quiet Enjoyment

Similar to the participants above, many of the participants in this study, found the period during the COVID-19 pandemic to be a time for quiet enjoyment of their baby and their new family as they were required to stay at home. Using the lens of feminist poststructuralism, we can see how these mothers were challenging social norms and expectations of new mothers. Challenging social ideals was a way of using their agency as they clearly articulated their beliefs about how being at home with their babies was very positive and for some a ‘blessing’. Many participants believed that this was a time for more personal and intimate family enjoyment. A time where the immediate family could not only enjoy each other but also their new baby.

It’s been great … we have this opportunity to bond as a family and he [partner] is here for every moment during the newborn stage! It has been amazing not having to worry about visitors coming and going and cleaning out home and me worrying about breastfeeding in front of others - instead we have a very relaxed atmosphere for everything!

As the above quote reveals, the participant believed that this was an opportunity to connect with her son in a way that would not have been possible outside of the COVID-19 pandemic. It was a time without the worry about visitors and the social expectations that are often placed upon new parents to be perfect and have everything altogether, such as keeping a clean and immaculate house, while tending to the needs of their new baby. It was a time to let go of the many worries of being a new mother that stem from social and cultural discourses of new motherhood, such as breastfeeding discourses. Social breastfeeding discourse often positions public displays of breastfeeding to be inappropriate and can create feelings of discomfort for the general public as well as mothers who are breastfeeding [37]. The participant revealed that isolation during COVID-19 allowed her to put aside such worries and pressures relating to breastfeeding so that she could relax. For many participants, as exemplified here, isolation was constructed as a time to be removed from societal pressures and to bond as a family. 

### 3.4. The Blessings of Learning

The COVID-19 pandemic not only provided an opportunity for intimate family bonding and freedom from the social pressures of new parenthood, but it also provided time for many participants to learn. Their learning was focused on their new baby as well as learning about themselves as new parents. For example, one participant believed that they [parents] “had this time to support each other and see each other grow as parents”. This time was valued as a time in which they could come to understand their new identities as parents - a time for her and her partner to grow together in these new roles and identities. Other participants also noted similar experiences, as illustrated in the quote below:

We were able to spend so much time alone as a family, and constantly being with our new baby has made us able to learn so much about her and enjoy spending our time with her without any distractions.

The time without distractions was seen as a benefit of being a new family. For participants, isolation during COVID-19 gave their families the opportunity to learn about their new baby and each other. It was a time for enjoyment and coming to know and understand their baby without the interference of others. 

### 3.5. The Blessings of Bonding and Snuggles

Participants noted how the experience of COVID-19 created time for their family to bond and learn about each other. “I found it great to bond as a family. I’ve really learned what works and doesn’t work for us”. This participant believed that this period facilitated greater understanding of their family dynamics and individual roles. Time and space had been created so that they could figure out family strategies that worked and that did not work for them, without interference, judgement, or input from others. 

COVID-19 was also a time for the enjoyment of the simple pleasures of having a new baby that many times can often be overlooked in the attempt to be “super moms”. For example, another participant recognized that although following the requirements to stay at home was “extremely hard” it was also enjoyable, noting that she has “been loving all of the one-on-one time with my son, the snuggles, and the fact that there have been limited distractions”. During isolation, distractions from others was limited and in this less hectic space, some participants were also able to find time to touch, bond, and snuggle more than they would have otherwise. They were able to experience the blessing of snuggles and connections with their babies even within the difficult times of the pandemic. Snuggling, touching, holding, kissing, and hugging are all noted to be deeply involved with bonding and mothers are told through social discourse that such bonding is critical to experience as a ‘good mother’ [38]. 

However, the social construction of mothering and parenting in Western societies often produce knowledge and competing discourses about how to hold, touch, and be with one’s newborn, especially in public spaces amid the distractions of daily life and other people. Being out in public can interfere with the way parents interact with their newborn. Often within public spaces, certain types of bonding and touching are positioned as too intimate and inappropriate for others. This can create experiences of tension for some mothers as they try to bond with their babies in publicly appropriate ways [39]. For example, as previously described, it has been noted that some mothers experience feelings of embarrassment while breastfeeding in public places [37]. Parents continue to struggle with these competing discourses when making decisions about how to interact and bond with their babies and figure out what is right for themselves and their babies. Participants, however, found that during COVID-19 they were able to better navigate these competing discourses to experience intimate bonding with their babies. As highlighted, in the previous quote, the mother’s focus on the joy of snuggling is not just a simple act; her emotional connection and her desire to bond in the best way for her baby was evident in her experience. This demonstrated the participant’s value of shared, dedicated, and intimate time with her baby, physical connection and bonding, and respect for the sacred space that is shared between parent and baby. In their bonding, participants challenged public norms of distancing, as well as mainstream judgements and ‘distractions’. Such participants enacted their own agency and chose to enjoy their family space during COVID-19 for important snuggling.

### 3.6. The Curses

In this study, we define curses as a challenge, a cause of harm or misery, or something that negatively influences well-being. In addition to identifying many unexpected rewards and positive outcomes during this period of public health measures, new parents also identified many challenges. The majority of mothers in our study revealed that they experienced complex contradictions during this time. In contrast to the ‘Blessings’ there were many ‘Curses’.

### 3.7. The Curses of Isolation 

Many participants believed COVID-19 had been a blessing but also a nightmare for their family. As noted in the preceding section, the first participant contrasted being free from the pressure to be a “super mom” against feeling as if she was living a nightmare. Using strong sentiments, she said, “In bad moments I just want to cry because of the pandemic. I am so sad that I can’t share her [the baby] with anyone”. This mother believed that sharing the experiences of having a new baby was important and felt sadness that she was not able to do this on account of the public health orders in place. Feeling ‘so sad’ revealed the meaning this experience held for this mother and that it was very significant. This was further emphasized as the participant gave more context to her experiences.

We were supposed to fly to Alberta to see my family at the end of March but had to cancel the trip. This is my family’s first grandchild so it just breaks my heart they will miss her whole babyhood. I also feel so alone with the baby. I have nobody here to help me figure out what is normal or how to progress through these early days. Although people can video chat it isn’t the same. I just want somebody to be in the room with me and the baby to see the things she can do and help me with things.

While isolation created the opportunity to bond together and feel free from outside pressures, it also created concerns. This participant expressed that she felt like she was missing opportunities to share the joy of her baby with others, which also resulted in lost moments for her and her baby. In the above quote, the mother also expressed feeling a lack of support and that although there were other options, like video chat, they were not the same. It was important to have others physically in the room to help which was not possible during this time. Without such in person support she felt loss on what was normal or how to progress as a new mother. New mothers are often faced with many contradictory social discourses on how to be mothers and what is best for their babies. Many participants felt that if public health measures requiring that people stay within their household were not in place than they would be better able to access support from others to help them figure out what was “normal” for new babies (discourses of normalcy). Previous research has explored how mothers navigate the discourses of normalcy through networks and connections with other new parents [22,23]. However, these usual networks were generally not accessible during the COVID-19 pandemic. 

### 3.8. The Curses of Robbed Momemts

Welcoming an infant into a family is not only an experience for the new parents but is also a significant experience for extended family and friends. Feelings of loss as a result of not being able to be around other people during this time was shared by many of the participants. One significant finding was that parents felt “robbed” of the ability to share their baby with their extended family and close network. One participant noted that, although the peak period of the COVID-19 pandemic was a time for family learning and creating connections between herself, her partner, and her baby, it was also a time of negativity for her and her family. She stated that the pandemic “affected us negatively because as much as we love having our new baby all to ourselves, we are also longing to show off our new baby to our family and friends”. She had the desire to share her new baby with others outside of her immediate partner. She further clarified what missing the shared experiences with her friends and family meant to her.

This pandemic has robbed us of so many other things, things that may not seem important to others. We had a photo shoot booked to capture pictures of her as a newborn, which was also cancelled due to the pandemic. I know in the grand scheme of things that is not a huge deal, but it does take a toll when it’s something you were looking forward to.

She believed that many of the typical experiences of new motherhood, experiences she was looking forward to, had been robbed from her and it had “taken a toll”. Although she recognized that these may be little moments in the “grand scheme of things” they were nevertheless moments or experiences that she valued as being an integral part of being a new mother and changing family. For example, the act of creating a family portrait through a professional photographer was lost to her and could not be recaptured. Although future photographs will happen, the opportunity to have photographs of her, her baby, and her family captured at that specific time is forever gone. Her baby will never be at this stage of life again. 

### 3.9. The Curses of Limited Socialiation and Bonding 

Many of the participants said they were concerned that their babies would not be socialized properly. It was felt by many that the COVID-19 pandemic limited their ability to socialize their babies with people, including family, friends, and other babies. One participant said,

I am home with a now six-month-old. She is missing out on social interaction with family, friends, and other babies. I can’t take my child shopping or to meet with other moms for coffee. I worry she will be overly attached to myself and husband, as we are who she sees outside of driveway visits from grandparents.

Another participant also spoke about the dichotomy created by staying home. On one hand, she expressed that “It was much easier to get into a routine. Without the constant onslaught of visitors..”. However, she was also negatively affected, evidenced with the following statement, “With that being said, it was VERY hard to not have the grandparents over to hold their new granddaughter. Many tears were shed behind panes of glass”.

In addition to the little moments that were lost and lack of socializing, another participant also believed she lost precious alone time with her baby, stating that although her family grew closer together she was also “sad that (she) was missing that alone time with my daughter”. Time to be alone with her baby, without her partner or others, was valued by this participant. She believed that as a new mother it is necessary to bond with your baby alone. The public health orders for family members of the same households to stay together prevented this participant from experiencing alone time with her child. The participant mourned this missed alone time to bond with her child.

To understand this mother’s experience, we need to look more closely at the meaning of alone time with her daughter. How had this ideal been constructed for this mother? Did she believe that she should be the main caretaker of her baby with the co-parent/father not so close? This can be viewed as a socially constructed discourse informed by heteronormative stereotypes of the roles of mothers and fathers [40]. Such discourse, also rooted in hegemonic gender binaries, often position women in the primary role of nurturer and the parent to bond with newborns. However, it is also possible that this mother valued time alone because it allowed her to more fully, holistically connect with her baby in a way that was unique to her and honoured her identity as a new mother. Following public health orders of staying at home brought out unique feelings about bonding and spending special time with one’s baby. In contrast, other mothers spoke about the importance of their partners being able to spend time with their babies. Time that otherwise may not have happened without the isolation measures put in place as a result of the pandemic.

### 3.10. The Blessing and Curses of Partners 

Experiences with partners was also discussed by many participants as a result of changing work habits and family circumstances, as exemplified by one participant’s comment, “since COVID-19 occurred, my husband has been working from home. While I continue to be on my maternity leave, balancing his work at home has been a little bit of an adjustment”. Experiences with partners were often viewed by many participants as both blessings and curses. This was reflected in the words of one participant, “my husband is home constantly which is positive and negative”. The blessing of partners included more support and help with the daily activities of their homes, including cooking and cleaning, as well as baby duties, such as changing diapers and holding. As another participant clarified this sentiment, saying, “he is available to help with diaper changes and to hold the baby when I need a quick break, but it isn’t what I pictured for my maternity leave”. The curse side of having partners home often took the form of more chores and mess relating to them being constantly at home. As another participant discussed, “my husband has been more supportive as he’s home more but there is also more to do since there are more meals to cook and more messes to clean and more activities to plan”. More chores, more messes, and more planning of activities were created for many participants as a result of their partners always being home during the pandemic. It was noted by several participants that the constant nearness of partners sometimes created certain tensions in their relationship. For example, one participant noted that “although having my husband home allowed him to spend more time with the kids, it increased stress between us we argued more”. This quote emphasized the experiences of participants who believed that the pandemic heightened stress and created strains within their personal relationships. Social and medical discourses often position family togetherness as critical during this time [6]. 

## 4. Discussion

According to Foucault [41], language, discourse, and knowledge are concepts that are interconnected to (re)produce social meanings and practices. Feminist poststructuralism positions language as structuring the way things are thought, and the way people act on the basis of those thoughts. Language is, however, set within historical contexts and as a result, is not stable nor does language represent a truth or one meaning. Language has multiple meanings that change depending on the social and political circumstances in which people live [32,41,42]. Discourse, however, moves beyond language to represent the interrelated systems of social meanings and practices “that systematically form the objects of which they speak” ([41], p. 49). Discourses are constantly being re-created as people collectively think and talk in different ways about the world. Language and discourses, therefore, (re)create knowledge and knowledge (re)creates the way people speak, come to know themselves, and shapes their values, beliefs, and practices. In other words, the subject. We can never fully understand all the influences that affect our subjectivity [42]. The subject is “written and overwritten through multiple and contradictory discourses” ([43], p. 275). The subject can be thought of as a palimpsest, a manuscript on which the writing has been partially erased to make room for other writings but with traces of the original remaining [43]. This metaphor illustrates how a subject is constantly in process and being shaped; written through multiple discourses layered upon and affecting each other. The subject is not ever blank. There is no pre-discursive self as one is never outside the influence of discourses [43].

This research revealed that there were many social discourses that shaped how the mothers experienced their babies and their wellbeing during the self-isolation period of the COVID-19 pandemic. Discourses of productivity for new mothers, discourses of bonding and connections during the postpartum period, discourse of touching, snuggling, and breastfeeding, discourses of normalcy, as well as heteronormative discourses of gender were all revealed through the analysis of participants’ responses. These discourses (re)shaped the experiences of the mothers reported in this study. The mothers’ experiences were seen as either positive blessings, negative curses, or, more often, seen as a complex interplay of blessings and curses, as highlighted in the results. Discourses of productivity, for example, created experiences for some mothers of freedom from the pressures associated with being a productive new mom, or as some participants said a supermom. This allowed them time to get to know their baby and time to enjoy living in the moment with them, a blessing of freedom. Discourses also created experiences that were negative or curses. For example, discourses of normalcy allow mothers to know and understand what is “normal” for their babies. Since the COVID-19 pandemic resulted in isolation many mothers felt that they were not able to connect with others to understand what is normal for babies. Participants also described the importance of special moments with babies, partners, and extended family. It is known that sharing and celebrating joys, moments, and successes together is vital not only to the health and wellbeing of families, but to the formation of collective and individual identities within the family [44].

Previous research has studied the experiences of both mothers and fathers during the postpartum, especially in the experiences of depression and mental stress [45]. In another study, it was found that mothers often expressed differences between their expectations of postpartum experiences, such as bonding, and their actual experiences [46]. It has also been previously reported parents often feel they need more support during postpartum under normal circumstances [45]. This research creates knowledge about the experiences of a group of new mothers during the COVID-19 pandemic. We can see that isolation created by public health mandates affected all participants in the study. While there were many similarities, there were also differences in how the limits and constraints of isolation affected each participant. This alerts us to the need to listen carefully to the unique relational experiences of mothers and families. Although the participants of this study were similar in many ways, consisting of birth mothers who were predominantly heterosexual, partnered white women within Nova Scotia, Canada, the findings can move beyond the local context and inform nursing practice holistically. Future studies could further explore the impact of COVID-19 isolation on the health of mothers and other family members through focus groups or observations. The postpartum time is a time of redefining family relationships, bonding, and changes in mood or mental health under the best circumstances, but our study suggests that these are heightened by mandatory public health measures of self-isolation during the COVID-19 pandemic, and informed by discursive considerations of postpartum.

The results provide deep insights into the way mothering can be understood within healthcare practices practice. Nurses and midwives are critical to influencing the way people experience their new babies. Literature suggests that supportive relationships with nurses during this period is crucial for new mothers [47,48]. We must attune ourselves as nursing professionals to recognize the complexity of experiences during the postpartum time and, in doing so, we will be able to provide more holistic service to parents both in usual times and under extreme circumstances, such as physical isolation and pandemics. By exploring both the positive and negative experiences, the blessings and the curses, of the participants, we can create an understanding that can inform nursing practice. We can develop strategies to help new parents to focus on the blessing of snuggling, bonding, and family connections, while also providing them with strategies to help with the curses.

## 5. Conclusions

This study provides a snapshot into the experiences of new mothers and their families during a pandemic, a time that was both a blessing and a curse for them. Their experiences reveal that following public health orders was neither a fully negative nor fully positive time for new families. It was a time during which complex contradictions constructed by competing social discourses created difficult dichotomies. The participants discussed the joy of family bonding and how they felt relief from the pressures of daily activities. Yet, participants also described the loss of these cherished moments and special experiences including lost opportunities to share their baby with friends and family. They also described unfulfilled expectations and hopes in terms of sharing important milestones in person versus via video chats. As part of compassionate health care practice, we should acknowledge the significance of these joys and losses to new parents. These moments of complexity need to be recognized, valued, validated, listened to, and accepted as families continue to navigate the changes related to the COVID-19 pandemic.
